# The potential connection between inflammatory bowel disease and Alzheimer’s disease: mechanistic insights and therapeutic implications

**DOI:** 10.3389/fimmu.2026.1910604

**Published:** 2026-08-07

**Authors:** Lei Peng, Ziyi Wu, Xinyue Yuan, Ce Zhou, Hao Fu

**Affiliations:** 1 Hospital of Chengdu University of Traditional Chinese Medicine, Chengdu, China; 2 Chengdu University of Traditional Chinese Medicine, Chengdu, China

**Keywords:** Alzheimer’s disease, glymphatic system, gut dysbiosis, inflammatory bowel disease, microbiota-gut-brain axis

## Abstract

Inflammatory bowel disease (IBD) and Alzheimer’s disease (AD) are two chronic conditions previously considered pathologically unrelated. However, accumulating epidemiological evidence suggests that patients with IBD may have an elevated risk. This review systematically analyzes the mechanistic links between IBD and AD, emphasizing the roles of gut microbiota dysbiosis, systemic and neuroinflammation, and glymphatic dysfunction. We propose a putative conceptual framework incorporating the ‘gut–glymphatic axis’ as a potential conduit through which IBD-associated microbial and immune perturbations may influence AD pathology. Key pathways include short-chain fatty acids, tryptophan metabolites, trimethylamine N-oxide, and bile acids, all of which modulate neuroinflammation and amyloid-β clearance. Additionally, circadian disruption and impaired aquaporin-4 polarity in the glymphatic system may represent novel links between intestinal inflammation and neurodegenerative changes. Preclinical animal models suggest that colitis exacerbates AD-like pathology via NLRP3 inflammasome activation, neutrophil infiltration, and microglial dysfunction. Natural bioactive compounds—such as ginsenosides, curcumin, baicalin, berberine, and magnolol—show dual therapeutic potential in both IBD and AD by targeting shared inflammatory and microbiota pathways. However, validated comorbidity models and clinical trials are lacking. This review provides a scientifically grounded basis for future mechanistic studies and integrated interventions for IBD–AD comorbidity.

## 1 Introduction

Inflammatory bowel disease (IBD) comprises a group of chronic immune-mediated intestinal disorders, principally Crohn’s disease (CD) and ulcerative colitis (UC). Although these two entities differ phenotypically and pathogenetically, they share core features of dysregulated mucosal immunity and persistent gastrointestinal inflammation (1–3). Epidemiological data consistently show a sustained increase in the incidence and prevalence of IBD across multiple regions, underscoring a growing global public-health burden with important consequences for healthcare resource use, long-term disease management, and socioeconomic well-being (4).

Alzheimer’s disease (AD) is a progressive neurodegenerative disorder characterized by two defining neuropathological lesions: extracellular amyloid-β (Aβ) plaques and intracellular neurofibrillary tangles formed by hyperphosphorylated tau (5, 6). These lesions contribute to synaptic dysfunction, neuronal loss, and progressive, largely irreversible cognitive and functional decline. Increasing evidence indicates that microglia and astrocytes, the resident immune-regulatory glial cells of the central nervous system, amplify neuroinflammation through pattern-recognition receptor-mediated innate immune activation and sustained release of pro-inflammatory cytokines and chemokines, thereby accelerating neurodegenerative progression (7). The gut microbiota has also emerged as an important regulator of microglial activation and AD-related neuroinflammation, while peripheral immune cells further contribute to the inflammatory milieu associated with AD. Taken together, these findings suggest that AD is not only a central nervous system disorder but also a systemic condition shaped by dynamic interactions among the gut microbiota, the intestinal immune environment, and inflammatory mediators.

In addition, natural bioactive compounds have attracted attention because of their multi-target pharmacological actions, relatively favorable safety profiles, and cost-effectiveness, making them promising therapeutic candidates for both AD and IBD.

In this narrative review, we systematically examine the mechanistic links between IBD and AD, with particular emphasis on how IBD-associated immune dysregulation, chronic systemic inflammation, and gut microbiota alterations may contribute to neurodegenerative pathology. On the basis of current evidence, we propose a putative conceptual framework incorporating the gut–glymphatic axis as a potential conduit through which IBD-associated microbial and immune perturbations may influence AD pathology may influence AD progression. We also critically evaluate the therapeutic potential of natural bioactive compounds in modulating pathological pathways shared by the two diseases, thereby providing a mechanistic rationale for integrated and mechanism-informed interventions targeting IBD-AD comorbidity.

## 2 Patients with IBD are at higher risk of AD

### 2.1 Epidemiological evidence linking IBD and AD

Accumulating epidemiological evidence suggests an association between inflammatory bowel disease and Alzheimer’s disease, implicating shared pathophysiological mechanisms, including chronic systemic inflammation, gut microbiota dysbiosis, and neuroimmune crosstalk (8–11). However, the literature is not entirely consistent, with some studies reporting an increased risk while others suggest a more nuanced or even contradictory relationship. A critical appraisal of this evidence is therefore essential.

A large-scale meta-analysis reported an altered risk of AD among patients with IBD; however, the effect estimate was presented as OR = 0.37 (95% CI, 0.14-1.00; p = 0.05), which requires cautious interpretation because the direction and magnitude of the association depend on the reference group and analytical model (11). This might be due to the fact that meta-analyses are prone to be influenced by publication bias, as well as heterogeneity in the adjustment for confounding factors such as study design, population characteristics, age, gender, and medication. Moreover, as mentioned later in this section, the majority of large-scale cohort studies from different geographical regions consistently indicate a positive correlation between IBD and the subsequent risk of dementia. Therefore, this single contradictory meta-analysis result should not be overly interpreted as evidence of a protective effect, but rather should be regarded as a signal of the complexity and heterogeneity inherent in the current epidemiological data. In contrast, a previous meta-analysis by Liu et al. also supported an association between IBD and dementia, reporting a significantly increased dementia risk across age and sex subgroups (12). This observation was further supported by a Danish population-based cohort study (13). In addition, a Korean cohort study showed that female patients (adjusted OR, 1.01; 95% CI, 1.01-1.02) and individuals aged 65 years or older (adjusted HR, 1.14; 1.04-1.25) with IBD had a higher incidence of AD and dementia than younger patients with IBD (8).

Overall, evidence from large cohort studies and meta-analyses supports a clinically meaningful association between IBD and an increased risk of dementia. These findings highlight the need to consider cognitive impairment during the early and integrated management of IBD.

### 2.2 Animal experimental studies on the association between IBD and AD

Experimental studies using AD mouse models further suggest that intestinal dysfunction may contribute to neurodegenerative pathology (**Table 1**). At present, the majority of studies have built upon the genetic-accelerated AD mouse model and added acute colitis induced by dextran sulfate sodium (DSS). However, this chemically-induced acute colitis cannot replicate the chronic and recurrent features of human IBD, which manifests as persistent mild inflammation and progressive tissue damage over years or even decades. Additionally, the transgenic AD model leads to rapid and supra-physiological levels of β-amyloid protein deposition, which cannot accurately represent the slow, multifactorial neurodegenerative process involved in sporadic Alzheimer’s disease. Within this framework, He et al. reported that DSS-induced colitis in aged C57BL/6J mice exacerbated neuroinflammation and cognitive decline through an NLRP3 inflammasome-dependent mechanism (30). This effect was absent in aged NLRP3-knockout mice, suggesting that NLRP3 deletion may attenuate DSS-induced cognitive impairment. In another DSS-induced model, AD mice showed markedly increased amyloid-β (Aβ) deposition compared with wild-type controls, accompanied by reduced CD68+ microglial activation, an indicator of impaired innate immune responsiveness (14). Similarly, in 5xFAD mice, systemic inflammation, reflected by elevated circulating pro-inflammatory cytokines, and gut microbiota dysbiosis were associated with increased amyloid deposition (31). Collectively, these studies suggest that intestinal inflammation may aggravate AD-like pathology, but the inherent limitations of the models used preclude direct extrapolation to the human IBD-sporadic AD comorbidity.

**Table 1:** Summary of animal model studies on the association between inflammatory bowel disease and Alzheimer’s disease.

Animal model	Induced condition	Key observation (outcome measures)	Relevance to IBD-AD link	Reference
Male wild-type C57BL/6 and male AppNL-G-F transgenic AD mice	DSS-induced colitis/IBD-like inflammation	DSS aggravated AD pathology: increased Aβ deposition, increased plaque, decreased microglia CD68, and impaired Aβ clearance.	Intestinal inflammation can aggravate AD brain pathology	(14)
Inducible intestinal epithelium-specific Psen1/Psen2 double-deficient mice	Tamoxifen (for gene deletion)	Rapid weight loss, spontaneous intestinal inflammation, epithelial barrier disruption, bacterial translocation, impaired Notch signaling, dysregulated differentiation; microbiota depletion rescues colitis but not weight loss; PSEN1 expression regulated in human IBD	Presenilins (Psen1/Psen2) are major genetic risk factors for Alzheimer’s disease (AD). Their critical role in gut homeostasis suggests that intestinal dysfunction may contribute to AD pathogenesis via gut-brain axis, and that IBD and AD may share common presenilin‑related pathways (e.g., Notch signaling, barrier integrity).	(15)
Male C57BL/6 wild type (WT) and AppNL-G-F mice (AD model)	DSS in drinking water (colitis-like); oral probiotic	Probiotic attenuates DSS colitis. In WT brain: probiotic plus DSS increases microglia, neutrophil elastase, 5hmC, decreases c-Fos. In AD brain: increases microglia, decreases c-Fos, increases Aβ plaques slightly; unique increase of TNFα and IL-2 only in AD mice.	Supports gut-brain axis: intestinal inflammation worsens AD pathology; probiotic modulates neuroinflammation and glial responses with genotype-specific cytokine changes (TNFα/IL-2 increase in AD).	(16)
Male/female AppNL-G-F (AD model) and wild‑type mice	DSS-induced colitis	Acute intestinal inflammation increases aggregated Aβ in AD mouse brains. scRNA‑seq shows increased neutrophils in the brain. Neutrophil depletion by antibodies suppresses the colitis‑induced Aβ accumulation.	Supports that acute colitis promotes AD pathology via neutrophil infiltration into the brain parenchyma. Neutrophil‑targeted therapies may reduce Aβ accumulation and prevent colitis‑associated increase in AD risk.	(17)
AD rats (D‑gal+Aβ) and IBD mice (antibiotics)	D‑gal/Aβ injection; antibiotics	OMO improves memory, reduces Aβ/Tau, modulates gut microbiota, alleviates IBD dysbiosis	OMO may be used as a prebiotic to improve AD-like lesions and has potential therapeutic value for neurodegenerative diseases.	(18)
PD mice (α-synuclein in 5-HT neurons) and DD mice (corticosterone stress)	α-synuclein overexpression in dorsal raphe serotonin neurons; corticosterone injection	Depression-like behavior, reduced intestinal motility; brain and ileum show downregulated miR-199a-5p/miR-219a-5p, upregulated miR-200a-3p, increased TNFα/IFN-γ/NFκB1, and altered glial response (Iba1, GFAP)	Identifies a conserved inflammation‑miRNA pathway (miR-199a-5p/miR-219a-5p/miR-200a-3p) as a common driver of PD, DD, and gut dysfunction along the gut‑brain axis. Targeting this triad may treat motor, psychiatric, and GI symptoms simultaneously.	(19)
Mice and small intestinal organoids	γ‑secretase inhibitors (pharmacological); antibiotics for microbiota depletion	Inhibitors induce inflammation of the small intestine and colon; microbial depletion can reverse this phenotype; epithelial structure destruction, inflammatory factor release; impaired cell differentiation and proliferation in organoids	γ-secretase-mediated Notch signaling is the key to intestinal epithelial homeostasis. Inhibition of this pathway can be used for the treatment of Alzheimer ‘s disease and cancer, but it can cause intestinal inflammation.	(20)
Adult wild-type C57BL/6J male mice	28‑day subcutaneous infusion of P. gingivalis LPS (oral) or E. coli LPS (gut)	Both LPS induce neuroinflammation, AD-like cognitive decline and pathology via microglial TLR4/TLR2 with intact blood‑brain barrier. Only E. coli LPS causes systemic inflammation.	Chronic LPS from oral (periodontitis) or gut (IBD) sources can trigger AD-like brain changes before blood‑brain barrier disruption. Supports peripheral chronic inflammation as a driver of late‑onset AD.	(21)
16‑month‑old wild‑type and NLRP3 knockout mice	DSS-induced colitis	DSS induces colitis, memory impairment, microglial activation, A1‑like astrocytes, impaired glymphatic clearance, increased amyloid plaques, neuronal loss; NLRP3 KO protects against all these deficits.	Colitis exacerbates age‑related neuropathology (glymphatic dysfunction, amyloid accumulation) via NLRP3 inflammasome. Targeting NLRP3 may prevent IBD‑induced cognitive decline and AD‑like pathology in aging.	(22)
Female mice	DSS-induced colitis	DSS increases β-galactosidase, senescence markers (p53, Bcl2, Cxcl1, Mcp1), inflammation (Ptgs2, Vimentin), and dysregulated miRNAs (miR-149-5p, miR-96-5p, miR-34a-5p, miR-30e-5p); Fisetin restores these changes and increases beneficial Akkermansia muciniphila.	Fisetin alleviates DSS colitis by targeting senescence and inflammation while restoring gut microbiota. Since chronic intestinal inflammation and senescence are linked to AD risk, this study provides a potential therapy for IBD that may also benefit AD prevention.	(23)
Rats; plus LPS‑treated RAW 264.7 macrophages	Single transrectal 4% acetic acid (colitis); oral Donepezil (5 mg/kg/day) for 14 days	Donepezil improves colitis‑induced cognitive deficits, reduces hippocampal inflammation/apoptosis/histopathology; alleviates colonic NF‑κB, TNF‑α, IL‑6, IL‑1β, MMP‑9, oxidative stress, Bax/Bcl‑2, caspase‑3; suppresses NO in macrophages.	Donepezil (an AD drug) ameliorates both experimental UC and its associated cognitive dysfunction via anti‑inflammatory, antioxidant, and anti‑apoptotic effects. Supports repurposing AChEIs for UC patients at risk of cognitive impairment.	(24)
TnfΔARE/+ mice (spontaneous TNF-α-driven CD-like inflammation)	3′UTR deletion of endogenous TNF-α gene	Crossing TnfΔARE/+ (CD-like colitis) with 5XFAD mice decreased Aβ deposition despite elevated peripheral TNF-α, with selective microglial (not astrocytic) activation, increased leukocyte/perivascular macrophage infiltration, and synaptic degeneration; APP and amyloidogenic enzymes unchanged.	Suggests peripheral TNF-α mediates gut–brain crosstalk and highlights context-dependent AD modulation via enhanced microglial/perivascular macrophage-mediated clearance, rather than reduced Aβ production.clearance.	(25)
Interleukin-10 knockout mice (spontaneous CD-like colitis)	Gene knockout (spontaneous)	IL-10 deficiency in APP/PS1 AD mice (with spontaneous CD-like colitis) promoted DAM-II microglial polarization, enhanced Aβ oligomer phagocytosis, preserved synapses and cognition, and increased Aβ clearance; IL-10–Stat3 knockdown endorsed phagocytosis.	Suggests IL-10–mediated anti-inflammation may paradoxically impair Aβ clearance, implying that rebalancing rather than simply boosting anti-inflammatory responses is relevant for AD; provides a model to dissect colitis–innate immunity–AD crosstalk.	(26)
CD4^+^CD45RB^high^ T-cell transfer model (immunologically mediated colitis)	Adoptive transfer of naïve CD4^+^CD45RB^high^ T cells into Rag1^–^/^–^ immunodeficient mice	CD4^+^ T cells infiltrate the brain in proportion to colitis severity, display Th17 phenotype (RORγt^+^), and associate with hypothalamic astrogliosis; RORγt-deficient T cells reduce brain infiltration.	Confirms T-cell-mediated gut–brain trafficking and links Th17-driven colitis to neuroinflammation and behavioral disorders via preferential migration of pathogenic RORγt^+^CD4^+^ T cells into the brain.	(27)
5xFAD mice + acute DSS colitis	2% DSS in drinking water (acute colitis)	Acute colitis induced microbial dysbiosis and systemic inflammation; monocyte infiltration into the brain; reduced cerebral Aβ deposition; increased Aβ efflux into bloodstream; increased IL-2 and IL-6 in hippocampus.	Demonstrates that acute intestinal inflammation can modulate Aβ pathology via enhanced monocyte infiltration and peripheral Aβ clearance; highlights complex, context-dependent effects of gut inflammation on AD progression.	(28)
Tg2576 mice + DSS colitis	DSS-induced colitis in young (presymptomatic) Tg2576 mice	DSS colitis anticipated the onset age of learning and memory deficits in Morris water maze; Firmicutes/Bacteroidetes ratio changes comparable to elderly Tg2576 mice (accelerated microbiota aging); reduced GFAP expression in hippocampus suggesting astrocyte “loss-of-function” profile.	Provides first evidence that colitis in young, presymptomatic AD mice accelerates cognitive decline; suggests gut inflammation may serve as an early trigger for AD-like behavioral deficits before plaque deposition	(29)

### 2.3 Critical evaluation of evidence and research limitations

Although large-scale meta-analyses and cohort studies consistently suggest an association between IBD and an increased risk of dementia, interpretation of these findings remains constrained by residual confounding, population heterogeneity, and differences in study design. In a large-scale analysis involving more than two million participants, a causal relationship was identified between IBD and Parkinson’s disease, whereas no evidence of genetic pleiotropy or a shared genetic architecture was found between IBD and AD (32). Subsequent genetic studies reporting no overlapping genetic loci between IBD and AD further supported this conclusion (33–36). Thus, although genetic evidence has not established a direct causal relationship between the two conditions, the observed epidemiological association is more likely to reflect immune-mediated and inflammatory mechanisms, offering important directions for future clinical and translational research (37). Also, it must be recognized that although inflammatory bowel disease-associated Alzheimer’s disease and sporadic Alzheimer’s disease share similarities in neuropathological features (such as Aβ plaques, and neuroinflammation), their etiologies are different. Sporadic Alzheimer’s disease is mainly driven by endogenous microglial dysfunction, while IBD-AD follows a trajectory from the periphery to the central nervous system, where systemic inflammatory mediators disrupt the blood-brain barrier and indirectly activate glial cells. Genetic studies have shown that the disease-causing loci of IBD and AD are mostly different, indicating that their epidemiological association is mainly mediated through non-genetic inflammatory pathways rather than shared genetic susceptibility (38). Moreover, IBD not only increases the risk of AD, but also significantly raises the risks of Parkinson’s disease, multiple sclerosis, and vascular dementia, suggesting that chronic intestinal inflammation acts as a driving factor for multiple central nervous system diseases through different molecular pathways (39). Meanwhile, aging is an established independent risk factor for AD, and emerging evidence indicates that aging may also aggravate IBD progression (40). In addition, IBD is closely associated with anxiety and depression, both of which may increase the risk of AD (41). Future studies should therefore rigorously control for these potential confounders to more accurately determine the independent contribution of IBD to AD risk.

## 3 Potential mechanisms linking IBD to AD

**Figure 1:**
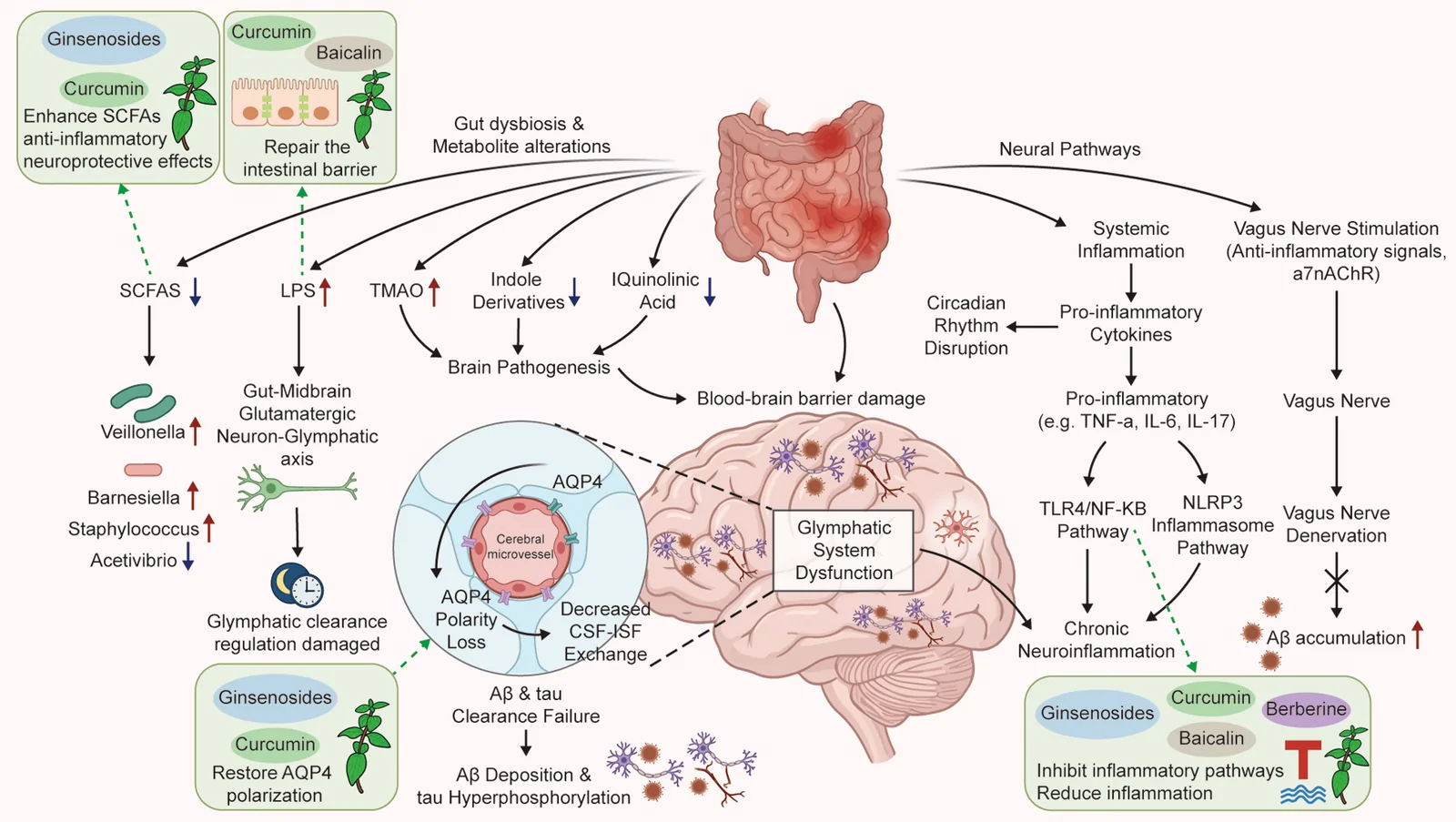
The role of the brain-gut axis in IBD pathogenesis and its bidirectional interactions with AD.

The gut-brain axis (GBA) represents a major bidirectional communication network through which IBD may influence AD pathogenesis. This reciprocal interaction is coordinated by multiple interconnected pathways, including pro-inflammatory cytokine release, immune-cell trafficking, autonomic nervous-system signaling, and microbiota-mediated regulation of neural and immune function (42). A recent study using a three-dimensional human intestinal-brain-vascular microphysiological system showed that AD-relevant stimuli delivered to the cerebral ventricles induced neuroinflammatory responses and compromised the structural and functional integrity of both the blood-brain barrier and the intestinal epithelial barrier. These findings support the existence of a bidirectional brain-gut-vascular axis in AD pathophysiology (43). Recent advances have refined this framework into the microbiota-gut-brain axis (MGBA) by formally incorporating intestinal microorganisms into the communication network (44). The microbiome has been proposed to communicate with the brain through pathways such as immune signaling, tryptophan metabolism, the vagus nerve, and the enteric nervous system (45, 46). Gut microbiota dysbiosis, chronic intestinal inflammation, and neurological dysfunction can disrupt GBA signaling and promote inflammatory immune activation, thereby worsening AD pathology. The autonomic nervous system is a key regulator of intestinal immune responses and constitutes a central component of the GBA. The vagus nerve represents the principal neural route of this axis. It conveys cholinergic anti-inflammatory signals that activate α7 nicotinic acetylcholine receptors on immune cells, particularly splenic macrophages and intestinal mucosal immune cells. This signaling suppresses the production of pro-inflammatory cytokines, including TNF-α and IL-6, and helps maintain intestinal immune homeostasis (47, 48). Rodent studies using vagotomy and vagus nerve stimulation have further suggested that modulation of vagal signaling may limit amyloid plaque accumulation and reduce AD-related risk (49, 50). Moreover, persistent intestinal inflammation can impair GBA function, promote inflammatory mediator production, trigger systemic inflammation, facilitate Aβ deposition, and accelerate the development of AD-like pathological features (51–53). The following sections therefore summarize the role of the brain-gut axis in IBD pathogenesis and its bidirectional interactions with AD (**Figure 1**).

### 3.1 Microbiota-derived metabolites

The human gastrointestinal tract contains a highly diverse and abundant microbial community composed of trillions of microorganisms. These commensal microbes contribute to host physiology by regulating immune function and modulating neurochemical signaling (54). Meanwhile, they can produce their own metabolites, precursor neurotransmitters, and immune regulatory factors, thereby influencing the development and function of the brain (55). The composition and functional capacity of the intestinal microbiota are shaped by host-intrinsic and environmental factors, including diet, lifestyle, and genetic background. Dysbiosis, defined as a persistent disruption of microbial community structure and functional balance, has been associated with adverse host outcomes such as endogenous toxin accumulation, chronic intestinal inflammation, and neurodegenerative disease (56, 57). Although patients with IBD and those with AD display distinct patterns of intestinal dysbiosis, emerging evidence suggests convergence in the direction of microbial shifts across the two conditions. Accordingly, recent studies have summarized gut microbiota changes in patients with IBD and AD. In both diseases, dysbiosis is commonly characterized by reduced bacterial richness, decreased abundance of beneficial short-chain fatty acid-producing bacteria, and increased abundance of pro-inflammatory taxa, especially Proteobacteria such as Escherichia/Shigella (37, 58–60). This IBD-specific dysbiotic profile—characterized by reduced alpha diversity, overgrowth of adherent-invasive Escherichia coli (AIEC), and depletion of butyrate-producing Clostridia—generates a sustained antigenic and metabolic challenge that may be more effectively transmitted to the brain than the comparatively milder dysbiosis associated with aging or sporadic AD (61).

#### 3.1.1 Short-chain fatty acids

Short-chain fatty acids (SCFAs) are the major metabolic end products of dietary fiber fermentation by the gut microbiota, with acetate, propionate, and butyrate being the most abundant and physiologically relevant forms (62). SCFAs exert biological effects mainly by activating G protein-coupled receptors and inhibiting histone deacetylase (HDAC) activity (63). Through HDAC inhibition, SCFAs can reduce the production of pro-inflammatory cytokines, including TNF-α, IL-1β, and IL-6, promote microglial polarization toward an anti-inflammatory M2 phenotype, and support angiogenesis and neurogenesis (64, 65). Although SCFAs generally exhibit neuroprotective properties, experimental colonization of germ-free mice with SCFA-producing bacterial strains increased Aβ plaque deposition (66). In germ-free AD mice, SCFA supplementation increased Aβ plaque burden to levels comparable to those observed in controls. Notably, in mice harboring a conventional microbiota, exogenous SCFA administration further aggravated Aβ plaque accumulation (66). These findings suggest that, at specific concentrations or under particular disease contexts, SCFAs may overactivate microglia, induce pro-inflammatory factor production, and indirectly promote Aβ formation (67).

#### 3.1.2 Tryptophan metabolism

Tryptophan (TRP) is an essential amino acid that cannot be synthesized endogenously and must be obtained from the diet (68). It is metabolized mainly through three pathways: the kynurenine pathway, the serotonin pathway, and the gut microbiota-mediated indole derivative pathway. Importantly, the intestinal microbiota regulates host neuroimmune homeostasis not only by directly generating bioactive TRP metabolites but also by modulating the expression and activity of host enzymes involved in TRP metabolism (69, 70). Gut microbiota can convert tryptophan into multiple indole derivatives, including indole-3-acetic acid (IAA), indole-3-aldehyde (I3A), and tryptamine (71). Indole-3-acetic acid, commonly abbreviated as IAA in this context, has been identified as an endogenous agonist of the aryl hydrocarbon receptor (AhR). AhR activation by IAA may exert neuroprotective effects by suppressing NF-κB-mediated neuroinflammation, strengthening intestinal epithelial and blood-brain barrier integrity through tight-junction regulation, and reducing oxidative stress by scavenging reactive oxygen and nitrogen species (72, 73). The kynurenine pathway represents the dominant route of tryptophan catabolism in mammals and is initiated by the rate-limiting enzymes indoleamine 2,3-dioxygenase (IDO) and tryptophan 2,3-dioxygenase (TDO), which are constitutively or inducibly expressed in host cells (71). Under chronic inflammatory conditions, pro-inflammatory cytokines strongly upregulate IDO expression, promoting the conversion of tryptophan into neurotoxic quinolinic acid. Sustained overactivation of this pathway contributes to excitotoxicity, mitochondrial dysfunction, and neuronal apoptosis (74, 75).

#### 3.1.3 Trimethylamine N-oxide

In recent years, increasing attention has been paid to the influence of dietary intake on the onset and progression of Alzheimer’s disease (76). Trimethylamine N-oxide (TMAO), a gut microbiota-derived metabolite, links the gut microbiota with the central nervous system through the gut-brain axis and may function as a key signaling mediator in AD pathology (77). In AD, elevated TMAO, derived from microbial metabolism of dietary choline, L-carnitine, and phosphatidylcholine, has been associated with two core pathological hallmarks: Aβ plaque deposition and tau hyperphosphorylation (78, 79).

TMAO may aggravate AD pathology through a multi-pathway signaling network that promotes Aβ aggregation and tau hyperphosphorylation. Mechanistically, TMAO has been reported to Aβ expression and promote tau hyperphosphorylation through the NLRP3 inflammatory pathway (80). In preclinical mouse models, TMAO robustly activated the PERK-eIF2α branch of the unfolded protein response in AD mice, resulting in impaired synaptic plasticity (81, 82). Notably, circulating TMAO levels increase with age, and this elevation has been reported to correlate with the progression and severity of AD-related neuropathology (83).

#### 3.1.4 Bile acids

Bile acids (BAs) are endogenous metabolites synthesized in the liver through enzymatic oxidation of cholesterol and subsequently secreted into the duodenum to facilitate lipid digestion and absorption (84). BAs primarily activate two major molecular targets, the nuclear hormone receptor farnesoid X receptor (FXR) and the G protein-coupled bile acid receptor 1 (GPBAR1, also known as TGR5), thereby regulating inflammatory responses and maintaining intestinal barrier integrity (85).

BAs are initially synthesized in the liver as primary bile acids and are then converted by the gut microbiota into secondary bile acids, including deoxycholic acid (DCA) and lithocholic acid (LCA) (86). These hydrophobic bile acids can compromise blood-brain barrier integrity by inducing tight-junction protein degradation and impairing endothelial cell function (87). After entering the brain, they may activate microglia and astrocytes, potentially increasing pro-inflammatory cytokine production; this sustained neuroinflammatory response has been hypothesized to contribute to AD progression (85). In the context of IBD-associated gut microbiota dysbiosis, levels of neuroprotective bile acids, particularly tauroursodeoxycholic acid and ursodeoxycholic acid, are often reduced, weakening their inhibitory effects on pathological tau hyperphosphorylation (88). Studies have further shown that elevated deoxycholic acid and lithocholic acid concentrations in cerebrospinal fluid and serum are associated with higher Aβ burden and poorer cognitive performance in patients with AD, strengthening evidence for a pathophysiological link between bile acid dysregulation and AD progression (89).

### 3.2 Immune/inflammatory pathways

The systemic inflammatory profile in IBD is characterized by elevated IL-6, TNF-α, and IL-17—cytokines that are not merely transiently upregulated but persistently elevated even during clinical remission, reflecting ongoing subclinical mucosal inflammation. This IBD-specific chronic inflammatory state differs qualitatively from acute or infection-driven inflammation in its duration, amplitude, and capacity to induce central nervous system effects. The following mechanisms illustrate how such IBD-derived inflammatory mediators may promote AD pathology.

#### 3.2.1 Cytokine signaling and microglial activation

IL-6, IL-17, and TNF-α are key pro-inflammatory cytokines involved in IBD pathogenesis (90, 91). These cytokines can activate microglia in the central nervous system, facilitating Aβ accumulation and tau hyperphosphorylation (91–93). A study reported that TNF-α inhibitor therapy in patients with IBD was associated with a reduced incidence of AD among individuals with CD and UC. This finding provides interventional evidence supporting the importance of TNF-α signaling in AD pathogenesis in the context of IBD (94). Furthermore, elevated IL‑6 and IL‑17 may compromise the blood–brain barrier, enabling peripheral immune cells and inflammatory mediators to enter the brain (95).

#### 3.2.2 TLR/NLRP3 inflammasome

Toll-like receptors (TLRs) and NLRP3 are also considered to play important roles in both AD and IBD (96, 97). After ligand binding and activation, TLRs initiate downstream signaling cascades that activate the NLRP3 inflammasome and increase the expression and secretion of pro-inflammatory cytokines, thereby amplifying inflammatory responses locally in the intestine and systemically (98). In addition, NLRP3 inflammasome activation in the brains of patients with AD may be potentiated by gut-derived inflammatory signals, forming a pathogenic cascade from gut inflammation to systemic inflammation and ultimately to neuroinflammation (99).

#### 3.2.3 NF‑κB and JAK/STAT signaling

NF-κB and JAK/STAT signaling are extensively involved in cellular inflammatory responses, both pathways have recently been recognized as shared mechanisms in IBD and AD. In AD, activation of the NF-κB pathway is thought to promote chronic neuroinflammation, in part through its effects on microglial function (100). Similarly, in IBD, excessive intestinal NF-κB activation may drive the production of pro-inflammatory cytokines such as IL-1β and TNF-α, which may facilitate the passage of inflammatory mediators across the blood-brain barrier into the brain (101). In AD, excessive JAK/STAT activation aggravates microglia-mediated neuronal injury and promotes pathological protein aggregation, contributing to disease progression (102). In IBD, inflammatory mediators activate JAK/STAT signaling and regulate transcription of genes involved in immune control and inflammation (103). Beyond these well-established signaling mediators, emerging evidence has identified interferon-induced transmembrane protein 3 (IFITM3) as a novel molecular link bridging intestinal inflammation and neuroinflammation. In a DSS-induced chronic colitis model, IFITM3 expression is markedly upregulated in both the colon and hippocampus. This upregulation activates the NF-κB signaling pathway, triggering downstream neuroinflammatory cascades and ultimately contributing to cognitive impairment (104). These shared signaling nodes offer potential targets for dual-disease intervention.

#### 3.2.4 Th17/Treg imbalance

The roles of pro-inflammatory cytokines, including IL-6, IL-17, and TNF-α, in driving systemic inflammation have been extensively documented. Emerging evidence further indicates bidirectional immunological crosstalk between adaptive immune cells in IBD and AD (105). In the intestinal tract, homeostasis depends on a finely regulated balance between Th17 and Treg cells; chronic inflammation disrupts this equilibrium and promotes pathogenic immune responses (106, 107). In the brains of patients with AD, levels of Th17-associated cytokines, such as IL-12 and IL-23, are elevated, promoting differentiation of Th17 cells into IFN-γ-producing cells. Intestine-derived Th17 cells may then enter the central nervous system, further aggravating Th17/Treg imbalance and establishing a positive feedback loop (108, 109).

### 3.3 Glymphatic system

The glymphatic system is a specialized brain-wide waste-clearance pathway (110). It enables cerebrospinal fluid (CSF) to enter the brain parenchyma along periarterial spaces, where it exchanges with interstitial fluid (ISF) through aquaporin-4 (AQP4) water channels enriched on astrocytic endfeet. The resulting fluid mixture, which contains metabolic waste products, is subsequently cleared through perivenous drainage routes (111). Disruption of AQP4 polarization weakens glymphatic clearance, promotes the accumulation of neurotoxic proteins such as Aβ and tau, intensifies neurodegenerative cascades, and may thereby increase the risk of AD (110, 112–114). The gut–glymphatic axis refers to the ability of gut-derived microbial metabolites, inflammatory cytokines, and immune signals—transmitted through systemic circulation—to modulate glymphatic clearance. Within this framework, the glymphatic system acts as a downstream effector, whose functional status is influenced by upstream intestinal inflammation. The following subsections examine how the metabolic and inflammatory perturbations described above may converge to compromise glymphatic function, thereby providing a mechanistic link between intestinal inflammation and impaired brain waste clearance.

#### 3.3.1 Circadian rhythm and glymphatic system

The gut microbiota can influence glymphatic system function through multiple interconnected pathways. A healthy intestinal microbiota produces short-chain fatty acids (SCFAs) and neuroactive molecules such as serotonin and melatonin (115). These metabolites may reduce neuroinflammation, improve sleep quality, and support synaptic plasticity, all of which are important for memory consolidation (63, 116). By contrast, dysbiosis can heighten systemic inflammation, disturb normal glymphatic activity, weaken waste-clearance efficiency, and promote the accumulation of toxic proteins in the brain, ultimately contributing to cognitive decline (115, 117). Because glymphatic clearance is most active during sleep, circadian rhythm integrity is essential for maintaining effective brain waste removal (118). The composition and metabolic activity of the intestinal microbiota also follow diurnal rhythms, suggesting that microbial oscillations may help regulate immune and metabolic disturbances caused by circadian disruption (119). *In vivo* magnetic resonance imaging studies further showed that colonic inflammation in murine colitis models disrupts circadian regulation, remodels cerebrospinal fluid distribution and clearance pathways, and promotes metabolic waste accumulation in the brain parenchyma (120). These findings suggest that intestinal inflammation may impair the glymphatic system through circadian rhythm disruption and reduced clearance capacity, thereby creating a neurobiological environment that favors the early development of neurodegenerative disorders, including AD.

#### 3.3.2 Metabolites and the glymphatic system

Beyond circadian regulation, microbial metabolites may also influence glymphatic function. A Mendelian randomization study identified a statistically robust causal association between two bacterial genera (Veillonella and Barnesiella) and the glymphatic-function biomarker ALPS. Subsequent transcriptomic and spatial enrichment analyses showed that genes associated with lymphoid-like structures were significantly enriched in the midbrain, predominantly in glutamatergic neurons, with an enrichment proportion exceeding 40%. These findings support a proposed neuroimmune-microbiota axis involving the gut microbiota, midbrain glutamatergic neurons, and lymphoid-like structures (121). Chronic low-grade inflammation is frequently accompanied by changes in gut microbial composition. Emerging evidence suggests that reduced Acetivibrio abundance and increased Staphylococcus abundance may impair lymphoid-like system function by modulating circulating tetradecyl diethanolamine, a bioactive metabolite, thereby contributing to cognitive decline (122). The gut microbiota produces diverse metabolites that can cross the intestinal barrier and, under certain conditions, the blood-brain barrier, thereby regulating neuroinflammation and glymphatic activity either directly or through systemic signaling. Among these metabolites, SCFAs are well-recognized anti-inflammatory mediators that attenuate neuroinflammatory responses and help preserve glymphatic clearance. Butyrate, in particular, suppresses excessive microglial activation and downregulates pro-inflammatory cytokine expression. However, microbial dysbiosis markedly reduces SCFA biosynthesis and may therefore compromise this endogenous neuroprotective mechanism (115, 123). Lipopolysaccharide (LPS), by contrast, is a prototypical pro-inflammatory pathogen-associated molecular pattern (124). When intestinal barrier integrity and blood-brain barrier function are disrupted, LPS can enter the central nervous system. Within the brain parenchyma, LPS activates Toll-like receptor 4 and triggers downstream signaling cascades that lead to robust production of pro-inflammatory cytokines and mediators (124, 125). Chronic exposure to these inflammatory stimuli disrupts the polarized localization of AQP4 at astrocytic endfeet, impairs cerebrospinal fluid-interstitial fluid exchange, and consequently weakens brain waste-clearance capacity (126).

In summary, intestinal inflammation generates a distinct metabolic and immune milieu; gut-derived factors enter the circulation, creating systemic inflammation and compromising the blood–brain barrier; and these signals converge on the brain, where they activate glial cells, trigger NLRP3 inflammasome, and impair glymphatic clearance, ultimately promoting Aβ deposition and cognitive decline. This cascade highlights the gut–brain crosstalk as a potential therapeutic target for IBD–AD comorbidity.

## 4 Natural products targeting IBD and AD

Current treatment options for IBD include aminosalicylates, glucocorticoids, immunomodulators, biologics, and JAK inhibitors. Emerging evidence suggests that certain IBD therapies may influence the risk of AD. TNF-α inhibitors, for instance, have been associated with a reduced incidence of AD in patients with inflammatory diseases (127). A nationwide study of over 340,000 IBD patients found that TNF-α inhibitor use was associated with significantly lower odds of developing AD in both Crohn’s disease and ulcerative colitis (94). Thiopurines such as azathioprine have likewise shown a lower incidence of AD (128). In contrast, long-term glucocorticoid use may impair certain brain structures and specific cognitive functions, although its association with dementia risk remains complex and, in some studies, even suggests a potential protective effect (129). However, the existing evidence is predominantly observational and subject to confounding by disease severity. Currently, there is no systematic comparison of the specific impact of different drug classes on AD risk.

These knowledge gaps, combined with the limitations of current pharmacotherapies, provide a rationale for exploring alternative therapeutic modalities, including natural products. Natural products, including baicalin and berberine, may exert therapeutic effects in IBD and AD through anti-inflammatory, antioxidant, and gut microbiota-regulating mechanisms. Compared with conventional pharmacotherapies, these agents offer distinctive advantages, including multi-target pharmacological activity and a relatively favorable safety profile with fewer adverse effects. These properties support their potential application in the management of IBD-AD comorbidity (**Table 2**). The following section summarizes how representative natural products modulate IBD- and AD-related pathological processes, with particular attention to anti-inflammatory, antioxidant, and microbiota-dependent mechanisms.

**Table 2:** The common pathways and effects of natural products in IBD and AD.

Nature products	Signaling pathway	Effect on IBD	Evidence level	Signaling pathway	Effect on AD	Evidence level
Curcumin	TLR4/NF-κB	Reduce IL-1β, IL-6, TNF-α, restore Treg/Th17 balance, and enhance intestinal barrier integrity and repair (130).	*in vitro*, animal	NF-κB	Inhibit NF-κB-driven neuroinflammation, microglial activation, and Aβ-induced neuronal damage (131).	*in vitro*, animal
Curcumin	Nrf2	Scavenge ROS in intestinal inflammatory foci and protect epithelial cells from oxidative damage (132).	*in vitro*	Nrf2	Reduce brain oxidative stress, protect neuronal mitochondria, and attenuate cognitive decline (133).	animal
Ginsenoside Rg1	PI3K/AKT	Modulate gut microbiota to boost SCFA production and inhibit NLRP3 inflammasome activation (134).	animal	PI3K/AKT	Protect β cells and neurons via the PI3K/AKT pathway and reduce Aβ production (135).	*in vitro*, animal
Baicalin	PI3K/Akt	Via PI3K/AKT inhibition, it reduced LPS-induced TNF-α, IL-6, IL-1β and apoptosis, while increasing IL-10 in HT-29 cells (136).	*in vitro*	PI3K/Akt	Reducing apoptosis and oxidative stress significantly enhanced cell viability (137).	*in vitro*
Baicalin	TLR4/NF-κB	Remove ROS, restore mitochondrial membrane potential, increase anti-inflammatory IL-10, and decrease pro-inflammatory TNF-α, IL-6, and IL-1 (138).	*in vitro*, animal	TLR4/NF-κB	Suppress microglial neuroinflammation by inhibiting NLRP3 inflammasome activation and the TLR4/NF-κB pathway (139).	animal
Berberine	AMPK	Improve intestinal energy metabolism, regulate intestinal flora, and repair the intestinal barrier (140).	animal	AMPK	Activate autophagy to clear Aβ and suppress neuroinflammation (141).	animal
Magnolol	PI3K-Akt	Reduce IL-1β, IL-6, and TNF-α; Repair the intestinal barrier (142).	animal	PI3K-Akt	Inhibit neuroinflammation, amyloid pathology, and synaptic dysfunction, and improve cognitive deficits via PI3K/Akt regulation (143).	animal

### 4.1 Ginsenosides

Ginsenosides are the principal bioactive constituents of Panax ginseng and display substantial structural diversity. Representative protopanaxadiol-type metabolites include Rg3, Rh2, compound K (CK), Rk1, Rk2, and Rk3 (144, 145). Extensive preclinical and clinical evidence provides support for their multifaceted pharmacological activities, particularly their antioxidant and anti-inflammatory effects with limited clinical data available (146). However, several ginsenosides, especially Rb1, Rg1, and Rg3, have low oral bioavailability and require intestinal microbial metabolism to generate active metabolites, which can increase their bioavailability by approximately three- to five-fold (147). In an MPTP-induced mouse model of Parkinson’s disease, CK significantly reshaped gut microbiota composition by increasing beneficial taxa such as Bacteroides acidifaciens and reducing potentially pathogenic taxa, thereby supporting a microbiota-targeted therapeutic strategy (148). Because inflammation is a key pathogenic mechanism shared by IBD and AD, the anti-inflammatory effects of CK are particularly relevant. CK significantly reduces pro-inflammatory cytokine production by 40-60% through selective inhibition of the TLR4/NF-κB signaling pathway (147). In addition, ginsenosides, including Rh1, Rg3, Rg5, Rb1, CK, and Rg1, can suppress inflammatory responses by inhibiting NLRP3 and NLRP1 inflammasome activation (149). In AD mice, ginsenoside administration significantly reduced serum IL-1β and IL-18 levels and suppressed NF-κB pathway activation, indicating a strong anti-neuroinflammatory effect (150).

As discussed above, gut microbiota dysregulation contributes to both IBD and AD. Experimental evidence suggests that ginsenoside Rk2 not only attenuates inflammatory-factor expression and colitis-associated symptoms but also reshapes gut microbiota composition, enhances SCFA production, and restores intestinal homeostasis by rebalancing the Th17/Treg cell ratio (151). Chronic administration of ginsenoside Rk3 in rotenone-induced Parkinson’s disease mouse models similarly altered gut microbiota composition, enriched Lactobacillus reuteri and Clostridium species, increased colonic butyrate concentrations, and attenuated neuroinflammatory responses. Moreover, ginsenoside Rk3 protected dopaminergic neurons through dual mechanisms involving HDAC inhibition and JAK/STAT3 pathway activation (152). Together, these findings indicate that ginsenosides are promising bioactive compounds for integrated IBD and AD management.

### 4.2 Curcumin

Curcumin is a naturally occurring polyphenolic compound with well-documented anti-inflammatory, antioxidant, analgesic, and antibacterial activities (153). Recent studies have shown that curcumin can modulate the NF-κB signaling pathway in both IBD and AD (154, 155). In IBD, curcumin increases the enzymatic activities of superoxide dismutase and catalase, reduces lipid peroxidation, and suppresses apoptosis of intestinal epithelial cells, thereby helping preserve intestinal barrier integrity (156). In neurological disorders, curcumin activates the Nrf2 signaling pathway by promoting nuclear translocation, facilitating binding to antioxidant response elements, and increasing the transcription of downstream antioxidant enzymes (157). After sustained curcumin administration in an Aβ1-42 mouse model, superoxide dismutase activity in brain tissue was significantly increased, whereas levels of the lipid peroxidation marker malondialdehyde were significantly reduced, indicating attenuation of oxidative stress-mediated neuronal injury in AD (158).

### 4.3 Baicalin

Baicalin and its aglycone baicalein are the principal bioactive flavonoids isolated from the roots of Scutellaria baicalensis Georgi (159). Both compounds have well-documented pharmacological activities, including anticancer, anti-inflammatory, and antioxidant effects (160). In patients and experimental models of IBD, baicalin and baicalein can alleviate intestinal inflammation by inhibiting NF-κB activity and reducing the expression of pro-inflammatory mediators such as TNF-α, IL-6, and IL-1β (161). In a rat model of vascular dementia, baicalein attenuated hippocampal neuroinflammation and neuronal injury through suppression of the TLR4/NF-κB signaling pathway, leading to significant improvement in cognitive performance (162).

The intestinal microbiota is critically involved in the pathogenesis of both IBD and AD. Wogonin has been reported to modulate gut microbiota composition and alpha diversity, increase SCFA production, strengthen intestinal barrier integrity, and ameliorate intestinal histopathological abnormalities in IBD models and patients (163). Shi et al. demonstrated that baicalein not only attenuated neuroinflammation in AD mice but also modulated intestinal microbiota diversity and composition, reduced the relative abundance of pro-inflammatory bacterial taxa, and improved AD-associated cognitive deficits (164).

### 4.4 Berberine

Berberine is a naturally occurring isoquinoline alkaloid with well-documented pharmacological properties (165). It alleviates colonic inflammation by inhibiting activation of the NF-κB and NLRP3 signaling pathways, downregulating pro-inflammatory cytokines such as IL-1β, IL-6, TNF-α, and IFN-γ, and upregulating anti-inflammatory cytokines, particularly IL-4 and IL-10 (166, 167). Berberine also exerts neuroprotective effects through inhibition of the NF-κB pathway. In APP/PS1 transgenic mice, berberine administration significantly suppressed NF-κB pathway activation, attenuated oxidative stress and neuroinflammation, improved cognitive impairment, and reduced aberrant tau hyperphosphorylation (168).

### 4.5 Magnolol

Magnolol and honokiol are bioactive compounds derived from Magnolia officinalis and have been reported to possess strong anti-inflammatory and antioxidant properties (169). Honokiol inhibits NF-κB pathway activation, downregulates pro-inflammatory cytokines, including TNF-α, IL-1β, and IL-6, and suppresses inducible nitric oxide synthase and cyclooxygenase-2 expression, thereby attenuating DSS-induced colonic inflammation (170). In a gut-brain axis-oriented study, a herbal combination containing Magnolia officinalis and Psilostylis xanthii markedly reduced NF-κB p65 phosphorylation in the hippocampus of D-galactose-induced aging mice and concurrently decreased pro-inflammatory cytokine expression in both serum and hippocampal tissue (171).

Although substantial evidence supports the efficacy of these compounds in single-disease experimental settings, no systematic investigation has yet examined their therapeutic value in comorbid IBD-AD phenotypes, a clinically relevant condition characterized by bidirectional gut-brain axis dysregulation. Therefore, hypothesis-driven and mechanistically informed preclinical studies using rigorously controlled dual-disease animal models are needed to clarify pharmacodynamic profiles, define optimal dosing strategies, and assess translational feasibility before first-in-human clinical trials are considered for patients with concurrent gastrointestinal and neurodegenerative pathology.

## 5 Future perspectives

Despite growing evidence linking IBD to AD pathogenesis, several critical knowledge gaps remain that must be addressed to advance the field. At the core of this inquiry is the unresolved question of causality—specifically, whether chronic intestinal inflammation actively initiates or accelerates neurodegenerative processes, or whether the observed epidemiological associations are largely epiphenomenal, shaped instead by shared confounders such as aging, dietary patterns, and lifestyle factors. Distinguishing between these possibilities demands a methodologically rigorous approach.

Equally pressing is the need for more physiologically relevant comorbid animal models. Current models, which typically superimpose acute dextran sulfate sodium (DSS)-induced colitis onto rapidly progressing transgenic AD mice, fall short of capturing the chronic, relapsing-remitting course of human IBD or the slow, multifactorial trajectory of sporadic AD. To address this limitation, future model development should prioritize two complementary strategies. First, chronic colitis induction protocols—such as multiple DSS cycles or spontaneous colitis models like IL-10 knockout mice—should be used to better approximate the persistent inflammatory state characteristic of human IBD. Second, aged wild-type mice or animals carrying sporadic AD risk genes should be preferred over transgenic strains that drive supraphysiological amyloid deposition. Integration of both elements would enable robust mechanistic dissection of the gut–brain glymphatic axis, including dynamic changes in aquaporin-4 polarity and glymphatic clearance capacity over time. To move beyond descriptive associations toward mechanistic understanding, we propose two specific and testable hypotheses for future investigation. First, in chronic colitis models, sustained administration of microbiota‑modulating agents—such as probiotics or natural compounds like ginsenoside Rk2 or baicalin—may delay or prevent Aβ deposition and cognitive decline by reshaping gut microbial composition and attenuating systemic inflammation. Second, glymphatic clearance function, as quantified by contrast‑enhanced MRI or fluorescence tracing, may serve as a key mediator linking chronic intestinal inflammation to the accumulation of neurotoxic proteins in the brain. Testing these hypotheses would not only clarify causal pathways but also inform the development of mechanism‑based therapeutic strategies for IBD–AD comorbidity.

In addition to these experimental directions, several translational and epidemiological aspects warrant attention. Although natural products have shown dual efficacy in preclinical settings, no clinical trial to date has evaluated these compounds in patients presenting with concurrent gastrointestinal and cognitive symptoms. Similarly, the differential contributions of Crohn’s disease versus ulcerative colitis to AD risk remain largely unexplored and call for dedicated phenotype-stratified epidemiological and genetic analyses. Finally, the advent of multi-omics technologies—integrating metagenomics, metabolomics, and neuroimaging—offers unprecedented opportunities to identify predictive biomarker panels that could enable early, risk-stratified screening of IBD patients most vulnerable to subsequent AD.

## 6 Conclusion

In conclusion, accumulating evidence supports a mechanistic link between IBD and AD, primarily mediated through gut microbiota dysbiosis, systemic and neuroinflammation, and glymphatic dysfunction. The proposed gut–brain glymphatic axis provides a novel framework for understanding how intestinal inflammation contributes to AD pathology. Natural compounds including ginsenosides, curcumin, and baicalin show dual therapeutic potential by modulating shared inflammatory and microbial pathways. Future longitudinal studies, validated comorbid animal models, and integrated clinical trials are urgently needed to translate these mechanistic insights into effective strategies for early screening and personalized management of IBD–AD comorbidity.
